# Downscaling air temperatures for high-resolution niche modeling in a valley of the Amazon lowland forests: A case study on the microclima R package

**DOI:** 10.1371/journal.pone.0310423

**Published:** 2024-11-07

**Authors:** M. J. Pohl, L. Lehnert, B. Thies, K. Seeger, M. B. Berdugo, S. R. Gradstein, M. Y. Bader, J. Bendix

**Affiliations:** 1 Laboratory for Climatology and Remote Sensing, Department of Geography, University of Marburg, Marburg, Germany; 2 Department of Geography, Ludwig-Maximilians-Universität, Munich, Germany; 3 Ecological Plant Geography, Department of Geography, University of Marburg, Marburg, Germany; 4 Meise Botanic Garden, Meise, Belgium; AUM: American University of the Middle East, KUWAIT

## Abstract

The forests of the Amazon basin are threatened by climate and land use changes. Due to the transition towards a drier climate, moisture-dependent organisms such as canopy epiphytes are particularly affected. Even if the topography in the Amazon lowland is moderate, mesoscale nocturnal katabatic flows result from cold air production related to radiative cooling. From a certain level of mass the cold air starts to flow downslope towards the valley centers leading to temperature inversions. The resulting cooling in the valleys drives localized fog formation in the valleys at night. This correlates with high epiphyte abundance and diversity in the valleys, which is much less pronounced upslope. The underlying temperature dynamics are, however, not sufficiently included in coarse-resolution reanalysis models such as ERA5-Land. Since high resolution climate data are needed e.g. for proper niche modeling of locally distributed species such as canopy epiphytes, downscaling models such as microclima have been developed and include micro- and mesoscale effects. However, it is unclear how well the elevation-related diurnal course of air temperature can be simulated. Here, we test functions for downscaling coarse-resolution temperature data to high spatial resolution data implemented in the R-package microclima for the South American tropical lowland forests. To do so we compared microclima-downscaled ERA5-Land air temperature data with meteorological station data. We found that the microclima functions only properly detect 73 temperature inversions out of 412 nocturnal cold air drainage (CAD) events during the dry season study period and only 18 out of 400 during the wet season with default settings. By modifying default values such as the emissivity threshold and time frames of possible CAD condition detection, we found 345 of 412 CAD events during the dry season and 177 out of 400 during the wet season. Despite problems with the distinction between CAD and non-CAD events the microclima algorithms show difficulties in correctly modeling the diurnal course of the temperature data and the amplitudes of elevational temperature gradients. For future studies focusing on temperature downscaling approaches, the modules implemented in the microclima package have to be adjusted for their usage in tropical lowland forest studies and beyond.

## Introduction

The forests within the Amazon Basin are endangered by climate and land use change [1–4]. Moisture-dependent organisms, especially canopy epiphytes, are particularly vulnerable to the shift to a drier climate [5–7]. Although the topography of the Amazon lowlands is relatively gentle, mesoscale nocturnal katabatic flows-triggered by cold air formation due to radiative cooling-cause cold air to flow downhill into valley centres, leading to temperature inversions [8–10]. This cooling effect in the valleys promotes localised fog formation at night, which is associated with higher epiphyte abundance and diversity in the valleys compared to the less pronounced presence upslope [9]. However, these temperature dynamics are not well represented in coarse resolution reanalysis models such as ERA5-Land [11]. High-resolution climate data are crucial for accurate niche modelling of species with localised distributions, such as canopy epiphytes, leading to the development of downscaling models such as microclima that account for micro- and mesoscale effects [12–15]. The Tropical Lowland Cloud Forest (TLCF) of French Guiana has been subject to different studies regarding CAD events, fog and low stratus cloud (FLS) formation and canopy epiphyte diversity. Canopy epiphyte communities characterized by a high abundance and high species diversity have been located within valleys of French Guiana with high nocturnal FLS frequencies [8–10, 16, 17].

During the day, the ground absorbs the solar radiation and is heated. Generally, lower-lying areas such as valleys are warmed more than higher-lying areas. During the night, the ground emits the absorbed heat again. The ground cools down rapidly as a result of the nocturnal radiation. This cooling is transferred to the air. As valleys are surrounded by slopes and have less heat exchange with the atmosphere, the air within them cools down more slowly than at the upper slopes of the valley. Cold air masses form at the upper slopes and start to flow towards the valley if they reach a certain level of mass. These cold air drainage (CAD) flows reach the valley center and lead to strong cooling. The CAD flows accumulate in the valley over time and, if the flow accumulation is strong, grow upslope flooding the valley with cold air [18–20]. The result is a temperature inversion in which the valleys are colder than the upper slopes. The conditions for this effect are particularly favorable at night when there is little cloud cover and the wind speed is low [8]. A further drop in air temperatures to the dew point initiated by the CAD flows can finally result in fog formation. This fog often remains trapped in the low areas of the valley and can persist until the early morning hours when the air warms up again after sunrise, clearing the fog and removing the inversions [9, 20]. This niche for organisms such as canopy epiphytes depending on FLS as a moisture source located in valleys is particularly affected by the transition towards a drier climate [21–23]. In order to be able to spatially model this niche formed by mesoclimatic temperature differences, spatio-temporal high resolution temperature data are essential. Climatic conditions are often influenced by small- and meso-scale phenomena such as terrain structure and land cover. ERA5-Land [11] and ERA5 global reanalysis data [24] are commonly used model datasets on climatic conditions on a global scale with high temporal resolution. Due to the coarse spatial resolution of the different ERA data, different studies have already set the goal of downscaling these data to a finer resolution [25–29]. It is important to consider that the models are typically calibrated for a defined area of application. Use beyond this must be checked on a case-by-case basis [13]. To downscale spatially poorly resolved data to a finer resolution, tools such as the *microclima* package in *R* have been developed. The application of the *microclima* functions has been recently validated for temperate forest [30]. For the South American tropical lowland forests, the performance of the *microclima* modules has not been validated yet. Thus, in this study an evaluation of temperature downscaling results of ERA5-Land data using the *microclima* algorithms is performed. Since the *microclima* algorithms are not calibrated for the tropics, adjustments have to be made in the detection of nocturnal cold air drainage (CAD) events. The time-dependent continuous accumulation of cold air lakes in valleys cannot be modeled correctly by the *microclima* algorithms. Therefore, in this study, especially the diurnal enhanced temperatures within the valleys compared to adjacent ridges as well as the nocturnal temperature inversions are modeled.

## Study area and measurement setting

**Fig 1:**
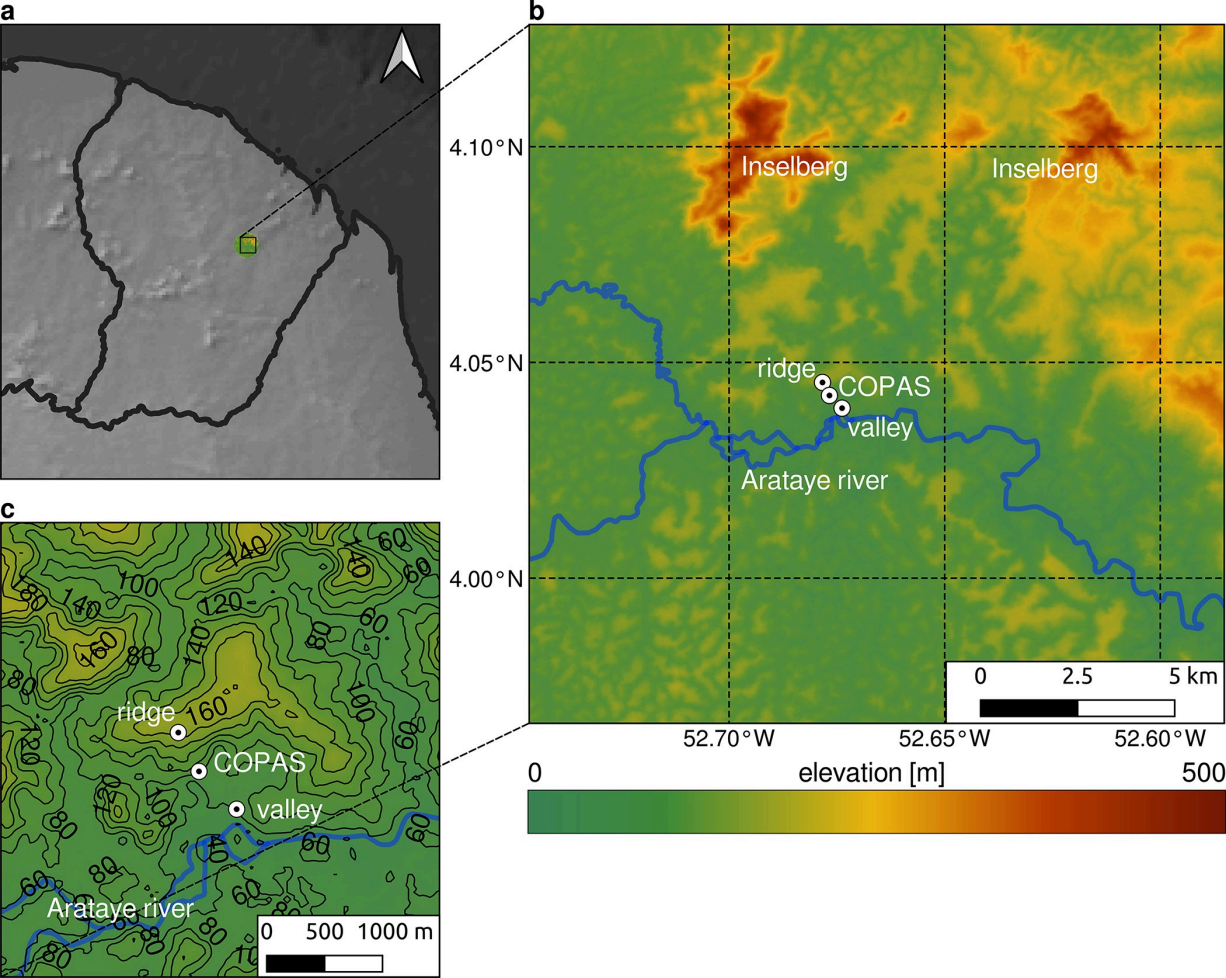
Large- and mesoscale localization of the study area and view on the terrain structure. **a** Large scale localization of the study area. **b** Mesoscale localization of the study area. **c** Detailed view on the terrain structure of the study area. Shuttle Radar Topography Mission (SRTM) Digital Elevation Model (DEM) serves as background map [28].

The study area is located in the predominantly pristine tropical forest of French Guiana and covers a total area of about 2,600 km^2^ (**Fig 1A**). The prevailing climate is tropical with slight fluctuations throughout the year. Cloud cover and rainfall vary strongly between the rainy and dry seasons. The rainy season with a dense cloud cover (mean = 84%) and high precipitation amounts (288 mm month^-1^) runs from January to June, while the dry season with a less dense cloud cover (mean = 55%) and low precipitation amounts (55 mm month^-1^) runs from July to December [31]. The terrain of the study area is characterized by a large number of small-sclae valleys, the Arataye River in the south and the inselbergs in the northeast [32–34]. An extraordinary high biomass gradient has been identified along this altitude gradient with a high species abundance and diversity in the valleys that is reduced in upslope direction. The strong cooling of the valley due to CAD events favours the frequent development of FLS in the valley, which supplies canopy epiphytes with moisture. This niche-development driving temperature gradient can be represented by the positions of the measurement stations.

As previously described by [8–10], nocturnal fog which is driven by nocturnal cold air drainage flows occurs at high frequency in the many small-scale valleys in this region. Visibility measurements are available for the wet and dry season at the Canopy Operating Permanent Access System (COPAS) located between the two temperature measurement positions. Visibility measurements serve as a predictor for FLS (visibility < 1km ≙ FLS) [35]. The frequent occurrence of FLS acts as a key driver for the development of a niche favorable for the settlement of epiphytes [36, 37]. Temperature and wind speed measurements are available at two stations and are used for validation in this study (**Fig 1B**, ridge, valley). They are placed along an elevational gradient with the ridge instrument at around 160 m a.s.l. and the valley instrument within the valley near the Arataye River at around 60 m a.s.l.

**Fig 2:**
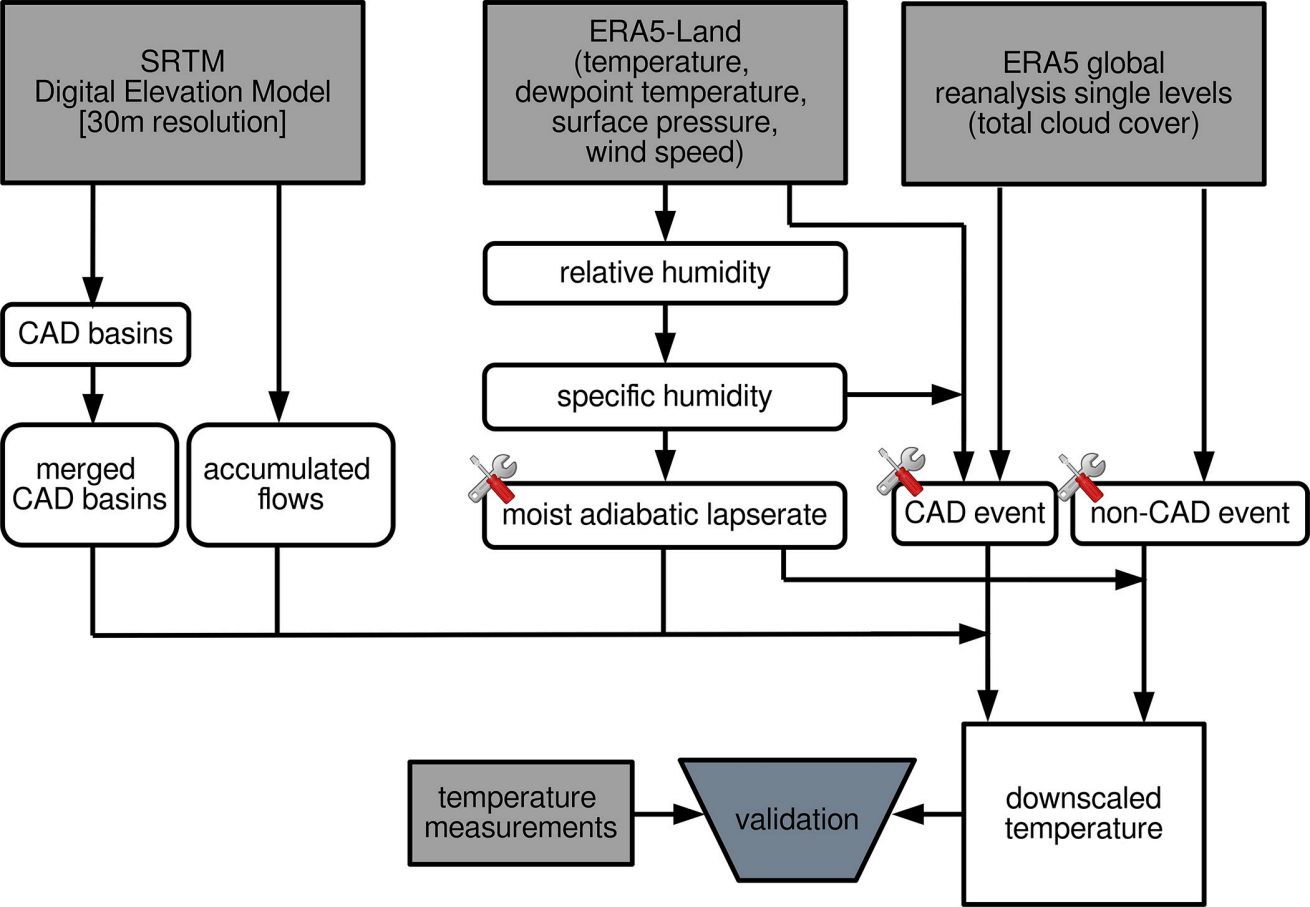
Processing scheme of data used (gray boxes), *microclima* processing (white boxes) and validation of the downscaled temperature. Tool symbols represent sections where adjustments were made to the *microclima* functions.

The aim of this study is to downscale ERA5-Land data with high temporal resolution (one hour) but coarse spatial resolution (~ 9km). For this purpose, a terrain model with a spatial resolution of 30m is used as target resolution and calculation reference. All ERA5-Land datasets are subject to a downscaling approach to 30m spatial resolution of this terrain model. To achieve this downscaling, functions from the R-package *microclima* are used (**Fig 2**). The functions use terrain and atmospheric information to derive small-scale changes in air temperatures caused by the aforementioned. In the first step of the *microclima* downscaling procedure, the constant terrain parameters CAD basins and flow accumulations are calculated. In a second step, the wet adiabatic lapse rate in each available timestep is calculated for the study area. Based on this and in combination with wind speed and cloud cover data a decision is made as to whether there is a CAD event (temperature inversion, T_valley_ < T_ridge_) or a non-CAD event (no temperature inversion, T_valley_ > T_ridge_). This results in two different downscaling procedures depending on the decision for each hour. Finally, the downscaled temperatures are tested against measurements at the ridge and valley positions (**Fig 1**). The two measurement positions are located in different grid cells in the target resolution. As a result, the two locations are assigned different temperatures after downscaling, which can be validated against the measurement data. Since the calculations in *microclima* are supplied solely from ERA5 land temperatures, all available temperature measurements can be used for validation.

## Data and methods

### Temperature measurements

We utilize mobile climate station data acquired above canopy from a ridge-valley setting with a ground distance of around 800 m and an altitudinal gradient of around 100 m (valley-ridge, **Fig 1C**) to validate the performance of the *microclima* downscaling functions in a tropical forest with respect to diurnal courses in air temperature. The installed instruments provide temperature measurements above canopy at 5-minute intervals which are aggregated to hourly averages to match the temporal resolution of the ERA5-Land data. Temperature measurements for the dry season in September and October (2007) and for the wet season in March and April (2008) are available. For the dry season there are 33 full days resulting in 792 total hours of which 53% (423) showed a temperature inversion (T_valley_ < T_ridge_, CAD events) and 47% (369) are non-CAD events (T_valley_ ≧ T_ridge_). For the wet season these are 31 full days resulting in 744 events of which 58% (435) have a temperature inversion (CAD events) and 42% (309) are non-CAD events.

### Digital elevation model, cold air drainage basins and flow accumulations

A Shuttle Radar Topography Mission (SRTM) Digital Elevation Model (DEM) [38] with a 30 m spatial resolution is used in the present study. The terrain model serves on the one hand as the spatial target resolution and on the other hand as a calculation reference for the different downscaling methods. The information on elevation, CAD basins and flow accumulations is derived from the DEM by means of *microclima* functions. The *microclima* function *basindelin()* is used to delineate hydrological basins where cold air drainage flows accumulate. The function analyzes the elevation information from a digital elevation model using a moving window. Grid cells that are higher than the central grid cell within a moving window are added to the basin of the central grid cell. This procedure is repeated until a grid cell elevation is below the already classified grid cells. If this occurs, the grid cell is classified into a new basin. The *‘basinmerge()‘* function joins adjacent basins. A threshold value can be set for the maximum height difference between neighboring basins. We test different thresholds of ten and 100 meters to clearly detect the effect of a minor and an extensive degree of merge. If a predefined threshold value is reached, adjacent basins are merged. Per default, basins are not merged and a default threshold for the usage of the *basinmerge()* function is not set and free to choose. Last, accumulated flows are derived by means of the *flowacc()* functions [13].

### ERA5-Land data and ERA5 global reanalysis data

In this study, the air temperature at two meters height, the dew point temperature and the wind components u and v from the ERA5-Land [39] data catalog in hourly resolution with a grid cell edge length of around 9 kilometers were used. Relative humidity is calculated from ERA5 temperature and dewpoint temperature values.

RH=100*exp((17.625*D)/(243.04+D))/exp((17.625*T)/(243.03+T))) (1)

with *RH = relative humidity in %*, *D = dew point temperature in °C*, *T = 2m air temperature in °C*.

Specific humidity is then derived by means of the *humidityconvert()* function from the *microclima* package, needed to calculate the moist adiabatic lapse rate:

SH=RH*0.6108*exp(17.27*T/(T+237.3))/P (2)

with *SH = specific humidity in kg/kg; T = 2m air temperature in °C; P = surface pressure [Pa]*.

In addition, total cloud cover data is extracted from the ERA5 global reanalysis [30] data. This information is needed to supply the cadconditions() function with information on the fractional cloud cover. For the calculations, the value of the single ERA5 global reanalysis grid cell which covers the study area is used.

### Detection of CAD conditions

To determine whether atmospheric conditions favor the development of a CAD event, the function *cadconditions()* can be used. The limiting factors in these functions are local time, wind speed and atmospheric emissivity. To analyze the performance of the *cadconditions()* function we tested how many measured temperature inversions are classified as CAD events. Additionally, we empirically adjusted the default limitations and thresholds according to temporal (local time) and atmospheric conditions (wind speed, fractional cloud cover) to measured values during measured temperature inversions.

First and by default, the *microclima* function sets a temporal restriction for the detection of CAD conditions between sunset and three hours after sunrise by default. The location dependent times of sunrise and sunset are derived by the function *suntimes()*. The times of sunrise and sunset are calculated dynamically depending on the location defined by latitude and longitude as well as on the time of the year. Since the study area is located close to the equator, sunrise is calculated for around 6 a.m. and sunset is calculated around 6 p.m. By default, CAD events can be detected from sunset until sunrise plus three hours. This results in possible CAD detections between 6 p.m. and 8 a.m. Local times in hours are assigned a classification as day (CAD not possible) or night (CAD is possible). A large number of measured temperature inversions (CAD events) occur at times when the *cadconditions()* function with default settings does not detect them. In order to be able to detect these CAD events as well, the detection window for the wet season after sunrise was extended by two hours (from 8 a.m. to 10 a.m.). For the dry season, the hours of possible CAD event detections were extended by two hours before sunset (from 6 p.m. to 4 p.m.).

Second, the functions query whether the wind speed is below a specified threshold. If this threshold is exceeded, CAD is not possible. The wind threshold in both functions is 4.5 m/s by default. To derive a threshold suitable for the ERA5-Land wind speed data used in the present study we used the *microclima* function *windheight()*. With this function, wind speeds at a specific height can be transformed to wind speeds at another height. We transformed the measured wind speeds during measured inversions at the two measurement stations into wind speeds at the ERA5-Land height level. For those wind speeds during temperature inversions we used the maximum for both seasons revealing 0.9 m/s during both seasons. This value represents the adjusted wind speed thresholds for the classification step as a CAD event in the *cadconditions()* function.

Finally, if the day and night classification reveals night and the wind speed threshold is not exceeded, the atmospheric emissivity is calculated within the *cadconditions()* function with:

e=Ps*(1−n^2)+0.976*n^2 (3)

with P_s_ = saturation pressure [hPa], n = fractional cloud cover [%]

The default emissivity threshold for the *cadconditions()* function is 0.5. If the threshold is exceeded by the result obtained from (3) the event is classified as a non-CAD event in microclimate, which might not be well-adapted for our study area. Thus, we empirically tested increased emissivity thresholds for both seasons to define an optimal value for CAD detection.

If the emissivity threshold is not exceeded and both other *cadconditions()* function tests (time and wind threshold) reveal a CAD event, the event is classified as CAD event. An event can only be classified as CAD, if all three requirements are met. In addition to the tests within the *cadconditions()* function we changed the *con* statement within the function from active to deactivated. If the *con* statement within the *cadconditions()* function is set active like by default, CAD events are only detected if CAD event conditions are detected for at least three consecutive hours.

### Model extent, elevational gradients and CAD basins

Not only the elevational gradient but the size of the study area is crucial for the calculation of CAD-related temperatures. The size of the catchment area is not set per default within the *microclima* functions. From wind speed measurements at the two measurement positions the maximum potential CAD catchment areas for an entire night with 16 hours of consecutive CAD occurrence was calculated by multiplying the mean wind speed by 16 hours. The maximum nocturnal CAD area is then defined on the basis of these calculations. CAD typically occurs during nights with low wind speeds or no wind. To classify atmospheric conditions regarding wind speed, we use the *windheight()* function from the *microclima* package to transform the ERA5-Land ten meter wind speeds to 1 meter wind speeds. The measurements from the transformed ERA5-Land data reveal an average wind speed of around 0.5 m/s on inversion nights, so that this speed is used as a potential propagation radius of CAD dispersion. This results in a maximum CAD catchment radius of 28.8 km. Additionally, we tested the effect of reduced wind speeds (0.25 m/s and 0.125 m/s) on the elevational gradients revealed. Additionally, a hydrological basin map is achieved using a DEM and the *basindelin()* function. By using the *basinmerge()* function, the basins are merged based on a height threshold. An optimized threshold value for the *basinmerge()* function is not defined. In principle, the function takes into account that CAD flows can pass from one basin into a neighboring basin if their height difference is small. However, since the effect of the threshold has a strong impact on the spatial characteristics of the result, we tested a low (10 meters) and a high threshold (100 meters) value.

### Correction of the microclima *lapserate()* function to derive terrain-dependent temperatures

Both during the day and at night, the lapse rate is decisive for the calculated altitude-dependent temperature. During non-CAD events the lapse rate is multiplied by the terrain height. As the lapse rate derived by the *lapserate()* function is always negative, grid cells at higher elevations have a lower temperature than grid cells at lower elevations. During CAD events, on the other hand, the lapse rate is multiplied by the difference between a grid cell and the highest point within its basin. Low-lying grid cells have a greater distance to the highest cell within their basin than higher-lying grid cells. This means that the lower a grid cell lies and the higher its distance to the highest point within the grid cell is, the greater is its cooling. The *lapserate()* function in the *microclima* package makes use of the specific gas constant of dry air (R_sd_) in both terms of the Eq (4). The correct equation for the calculation of the moist adiabatic lapse rate contains the specific gas constant of water vapour (R_sw_) in the second term of the equation. Thus we changed the *microclima* code by using the correct formula for the moist adiabatic lapse rate as follows:

lrd=g*(1+(h*rv)/(Rsd*(T+273.15)))/(1003.05+(0.622*h2*rv)/(Rsw*T+273.15)^2))*‐1 (4)

with lrd = moist adiabatic lapserate, g = Earth’s gravitational acceleration [9.8076 m/s^2], h = heat of vaporization of water [2501000 J/kg], rv = the mixing ratio of the mass of water vapour to the mass of dry air, T = reference temperature [K], R_sd_ = specific gas constant of dry air [287 J / kg*K], R_sw_ = specific gas constant of water vapour [461.5 J / kg*K]

### The process-based model KLAM21

The process-based cold air runoff model KLAM21 is another option for calculating CAD flows [40]. It is used to simulate the outflow of cold air in the lower atmospheric layers. The model is designed to analyze various aspects of cold air runoff, including the propagation of cold air flows in valleys and basins. The KLAM21 calculations provide the user with information on the cold air height [m] and the cold content [J/m^2^] of the cold air column. In this study, the results obtained from the KLAM21 model are used to compare a realistic, process-based CAD accumulation with the mircoclima drivers chosen like study area extent and included height information. The KLAM21 model was initialized with 0% and 50% cloud cover for representative model runs with good and moderate prerequisites for CAD flows. For both model runs we set the regional wind speed to 0.5 m/s from north-western direction. The models were run for a total of fifteen hours. The model requires a surface classification as an additional setting parameter. The entire terrain was classified as a forested area. Potential effects on the CAD propagation by the Arataye River were not taken into account at this point.

## Results

### Measured temperatures, original ERA5-Land temperature data and corresponding temperature differences

**Fig 3:**
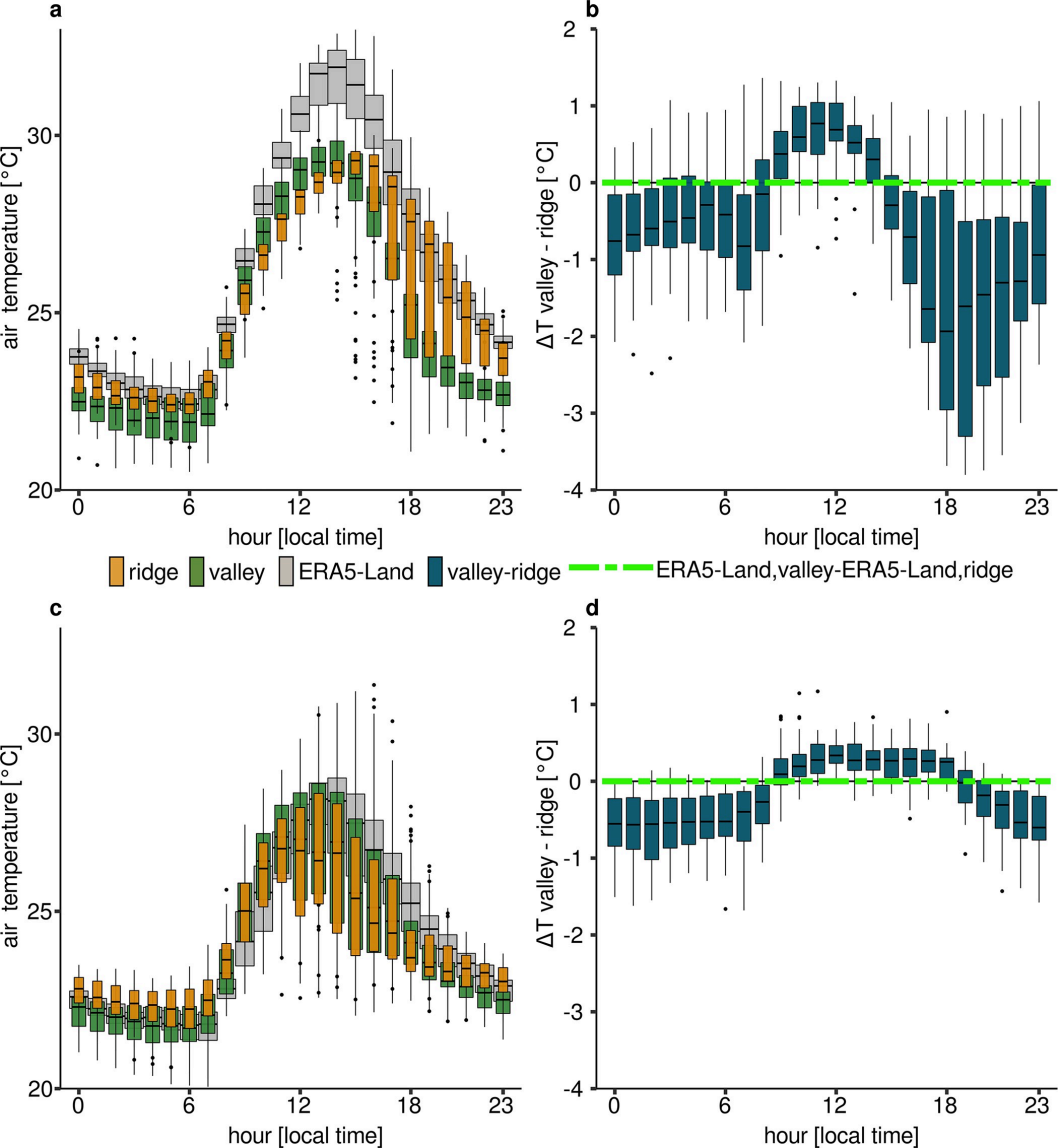
Temperatures and temperature differences at both measurement stations compared to ERA5-Land data. **a** Dry season measured temperatures and ERA5-Land temperature. **b** Dry season hourly temperature differences between valley and ridge compared to ERA5-Land temperature difference (green dashed line, both stations fall into the same ERA5-Land grid cell). **c** Wet season measured relative humidities and ERA5-Land humidities derived from temperature and dewpoint temperature. **d** Wet season measured temperatures and ERA5-Land temperature. **e** Wet season hourly temperature differences between valley and ridge compared to ERA5-Land temperature difference (green dashed line, both stations fall into the same ERA5-Land grid cell).

From the COPAS visibility measurements serve as FLS predictors. FLS occurs in 74% of nights during the dry season and in 96% of nights in the wet season. In both seasons, about 80% of FLS events are measured between midnight and sunrise. As the valley measurement position is located further down the same valley as the COPAS, the visibility measurements at the COPAS position also serve for the valley position where frequent FLS occurs. Previous analyses show that the FLS reaches the ridge station much less frequently [41]. Since the ERA5-Land data have a spatial resolution of around 9 kilometers both ridge and valley position are located within the same grid cell and share the same associated ERA5-Land temperature. During the dry season temperatures are generally overestimated by ERA5-Land (**Fig 3A**, RMSE_ERA5-Land, ridge_ = 1.8°C,; RMSE_ERA5-Land, valley_ = 1.8°C). In the dry season, however, the measured temperatures show a fairly regular scatter (**Fig 3C**, RMSE_ERA5-Land, ridge_ = 1.3°C,; RMSE_ERA5-Land, valley_ = 1.3°C). The dry season is characterized by a greater temperature amplitude within the diurnal course. Furthermore, the difference in temperatures along the altitudinal gradient is more pronounced than in the wet season (**Fig 3B and 3D**) [34]. The valley warms up as well as cools down faster and more strongly than the ridge, especially during the dry season. During the dry season, CAD flows during evening and early nocturnal hours lead to strong differences between the measured temperatures which are equalized by CAD accumulation towards the ridges later. The valley temperatures cool down fast from around 5 p.m. due to cold air drainage flows while the upper slopes are only affected by radiative cooling. The cold air mass accumulates in the valley over time and grows up the slopes, so that the upper slopes also cool down at around 1 a.m. which reduces the temperature differences between valley and slope. This diurnal terrain differences and thus, the mesoscale CAD processes are not resolved by coarse-resolution ERA5-land data.

### Pre-definition of model run extents and merging of cold air drainage basins

**Fig 4:**
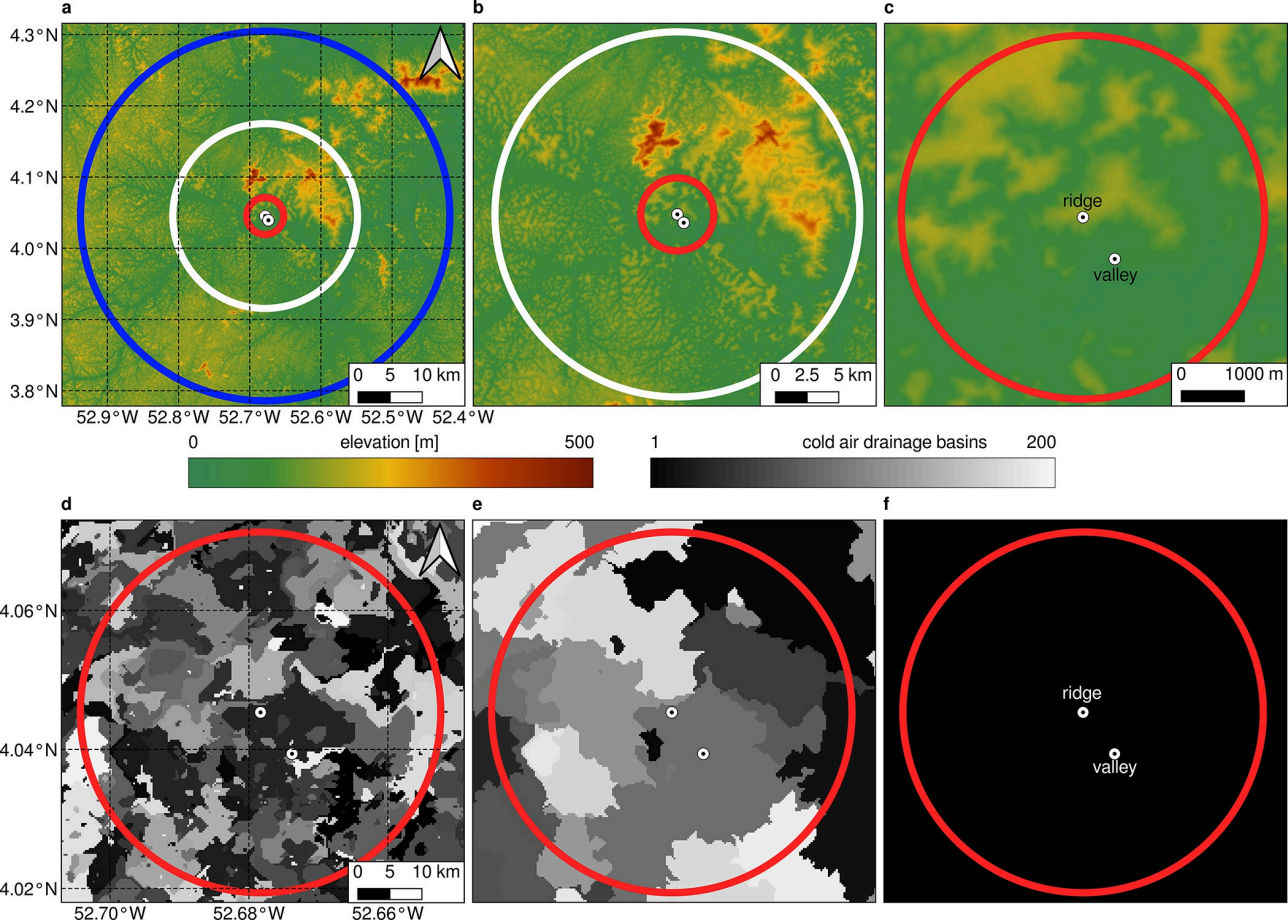
Study area extents and different degrees of CAD basin merge. **a** Maximum tested study area extent with a radius of 28,800 meters derived from 16 hours of consecutive CAD flows. **b** Reduced tested study area extent with a radius of 14,400 meters. **c** Reduced tested study area extent with a radius of 7,700 meters. **d** CAD basins within the extent from **c** with default settings. **e** CAD basins within the extent from **c** with a basin merge threshold of 10 meters. **f** CAD basins within the extent from **c** with a basin merge threshold of 100 meters. Shuttle Radar Topography Mission (SRTM) Digital Elevation Model (DEM) serves as background map [38].

The pre-definition of model run extents and the degree of basin merges is crucial for the results obtained with the *microclima* functions. By increasing the model run extent, the model uses an extended palette of height information (**Fig 4A–4C**). While comparably large model run extents (**Fig 4A**, **4B and Table 1**) incorporate the Inselbergs north of the measurement stations and serve a highest point in the model run extent of 482 meters, the Inselbergs are not included in the smallest model run extent (**Fig 4C**, **Table 1**).

**Table 1:** Effects of wind speed dependent study area extents and basin merge threshold on elevational gradients.

wind speed (m/s)	radius of study area extent [m]	basin merge threshold	total number of basins	highest point in basin of ridge and valley station	vertical distance to ridge station	vertical distance to valley station
0.500	28.800	0	21.729	181	123	15
0.500	28.800	10	1.051	181	123	15
0.500	28.800	100	1	482	424	316
0.05	5.760	0	1.215	181	123	15
0.05	5.760	10	75	181	123	15
0.05	5.760	100	1	482	424	316
0.01	2.880	0	339	181	123	15
0.01	2.880	10	26	181	123	15
0.01	2.880	100	1	423	365	257

On the one hand, as shown in **Table 1** the elevational gradient at both measurement stations (ridge, valley) increases with increased study area extents and increased merging of the basins. The height difference between the two stations (Elev_valley_-Elev_ridge_), on the other hand, remains constant regardless of the basin threshold used since both stations are located in the same basin. This results from the fact that the stations are close to each other and are captured in the same basin at default settings and threshold values of 10 and 100 meters. The calculation of their cooling therefore refers to the same highest grid cell within their basin. This causes the temperature difference between the two measuring points to depend solely on the lapse rate.

**Fig 5:**
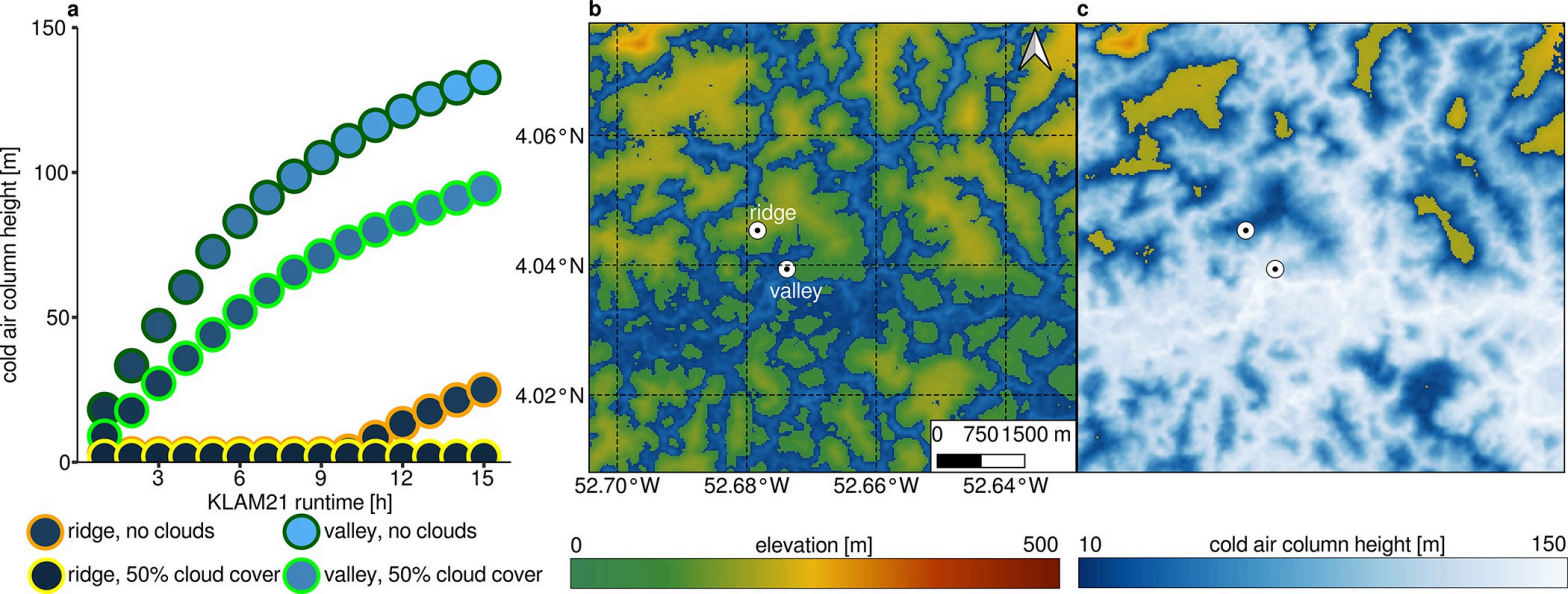
Cold air column height and spatial distribution of cold air calculated with KLAM21. **a** cold air column at ridge and valley position under different mean nocturnal cloud cover (0%, 50%) settings. **b** Spatial distribution of cold air distribution after one hour of CAD flows with 0% cloud cover. **c** Spatial distribution of cold air distribution after fifteen hours of CAD flows with 0% cloud cover. Shuttle Radar Topography Mission (SRTM) Digital Elevation Model (DEM) serves as background map [38].

Another option to determine the study area extent is the KLAM21 model. Compared to the *microclima* algorithm, the KLAM21 is able to model cold air mass accumulation within the valleys over time. Otherwise, it is not able to incorporate CAD conditions for single hours which is why the model is run for full nights under mean atmospheric conditions. Two different model runs reveal that the cloud cover is crucial for the results obtained with the KLAM21 model (**Fig 5**). If a completely cloudless sky (cloud cover = 0%) is assumed for the entire model period, the cold air column at the valley station grows strongly, especially in the first few hours. The growth weakens with increasing time (**Fig 5A**). At the ridge station, however, no cold air column grows in the first few hours. Only after the eleventh hour of the model run does the growing column of cold air in the valley reach the upper slopes and thus the ridge station. The spatial effect of the rising cold air masses over time is also displayed spatially (**Fig 5B and 5C**). In comparison, the cold air column with 50% cloud cover at the base station grows much more slowly and does not rise as high as in the previous model run. The ridge station is not reached by the cold air mass.

### Detected CAD conditions with default and modified settings

**Fig 6:**
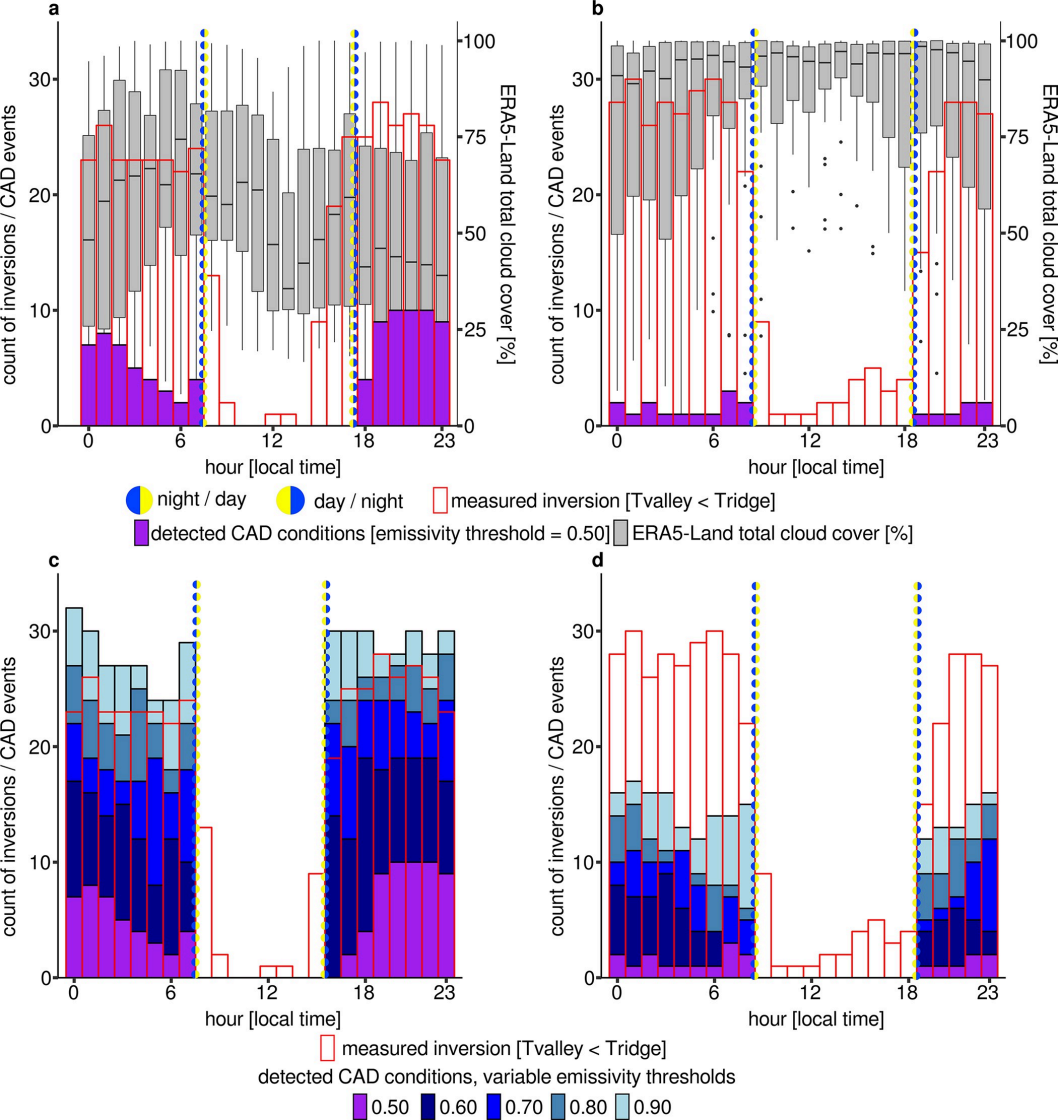
Cloud cover, measured inversions and detected CAD conditions. **a** Dry season with default settings. **b** Wet season with default settings. **c** Dry season with modified wind threshold, modified emissivity thresholds and modified daytime/nighttime classification. **d** Wet season with modified wind threshold and modified emissivity thresholds.

The *cadconditions()* function reveals a classification in hourly resolution, whether atmospheric conditions favor CAD or not. The following evaluation is based on the temperature gradients between valley and ridge. If the temperature at the valley station is lower than at the ridge station, there is an inversion. If an inversion is classified as an hour with favourable conditions for CAD flows, it is classified as a CAD event. Otherwise it is classified as a non-CAD event. In order to be able to model this inversion temperature gradient, an inversion must be classified by *microclima* as a CAD event. If the default emissivity threshold is used, only a few inversions are correctly detected as CAD events in both the dry and wet seasons (**Fig 6A and 6B**). During the dry season, there is less total cloud cover and more events are classified as CAD events. In addition, more inversions were measured in the wet season than in the dry season, even if their temperature gradient is not as strong. For both seasons, the proportion of correctly detected CAD events improves due to a distinct increase in the emissivity threshold. For the dry season, an emissivity threshold of 0.9 proves to be the optimal threshold value. In addition, the temporal detection window for CAD events before sunset must be extended in the dry season (**Fig 6A and 6C and Table 2**) CAD events. For the wet season, an emissivity threshold of 0.9 is still not set high enough. Almost 60% of CAD events are not correctly detected with an emissivity threshold value of 0.9 (**Fig 6B**, **6D and Table 2**).

**Table 2:** Results for the detection of CAD and non-CAD events compared by default and modified settings in the *microclima cadconditions()* function. Modified settings are enlarged possible hours for CAD detection during the dry season (4, 5 p.m.), an emissivity threshold of 0.9 and a wind threshold of 0.9 m/s.

season	settings	TP	TN	FP	FN	PC
dry	default	73	361	19	339	55
dry	modified	339	265	115	73	76
wet	default	18	341	3	382	48
wet	modified	168	310	34	232	64

### Humidity and lapse rate

**Fig 7:**
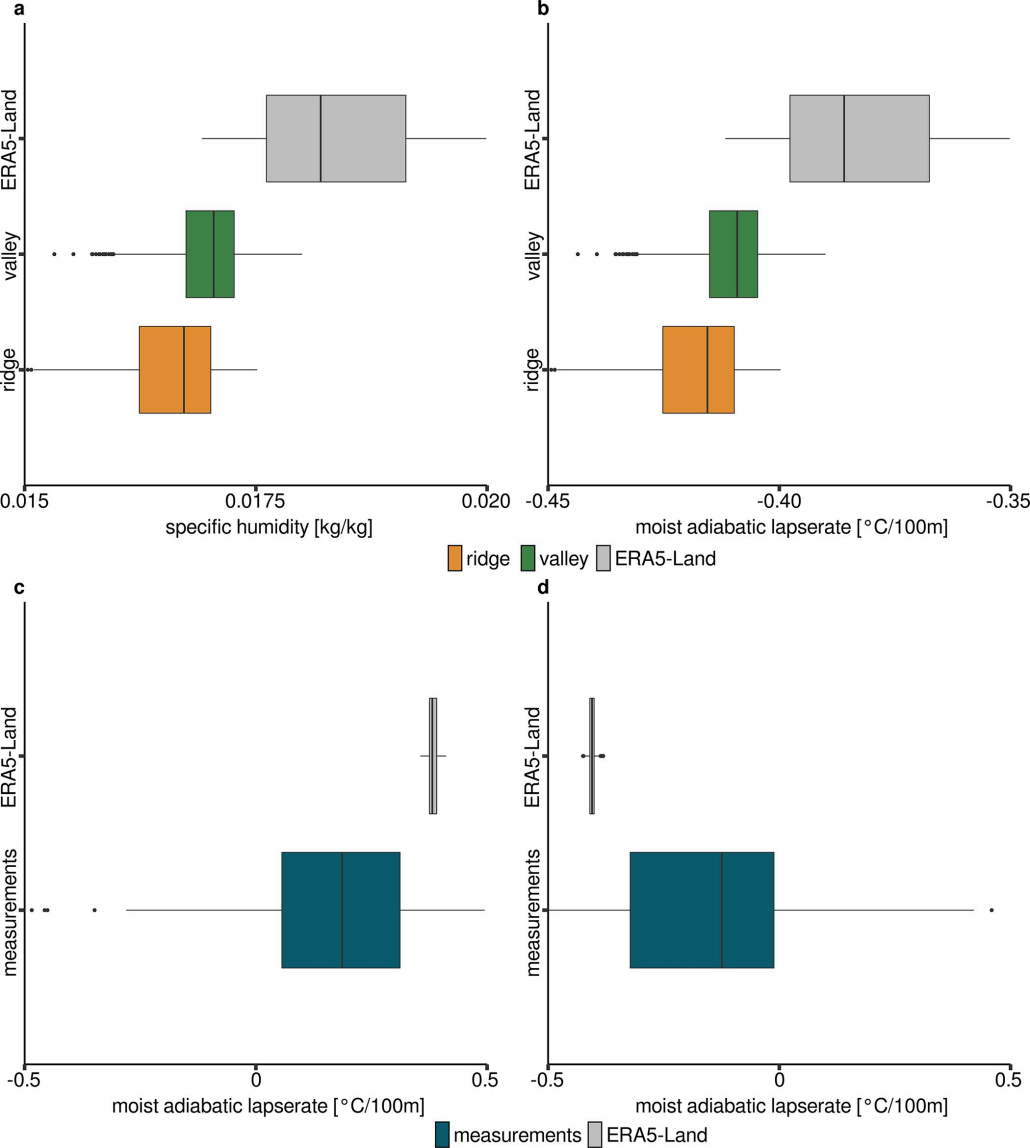
Comparison of modeled humidity and lapse rate with measured lapserate. **a** Specific humidity derived from measurements at the two measurement positions and ERA5-Land data. **b** Moist adiabatic lapse rate derived from specific humidities in **a**. **c** Measured lapse rate compared to ERA5-Land lapser ate (absolute values) during daytime. **d** Measured lapse rate compared to ERA5-Land lapse rate during nighttime.

From the available measurements of temperature and relative humidity at the valley and ridge position and original ERA5-Land data it is possible to produce comparable datasets of specific humidity. From this specific humidity data the lapse rate is derived in combination with pressure and air temperature by means of the *lapserate()* function (**Fig 7A and 7B**). The moist adiabatic lapse rate derived by the model and measurement values for humidity and temperature are compared to the in situ measured lapse rate (**Fig 7C and 7D**). The in situ lapse rate is the temperature difference between valley and ridge position divided by their vertical distance.

The specific humidity from the ERA5-Land data differs with an RMSE of 0.002 kg/kg from the measurements at the two measurement positions (**Fig 7A**). The deviations between the model data and the measured data are therefore marginal. Consequently, this is also apparent in the calculated values for the wet adiabatic lapse rate. The difference in the moist adiabatic lapse rate is very small at an RMSE of 0.02°C/100m. The measured lapse rate deviates considerably from the modeled values. The deviation between the modeled and measured lapse rate is 0.7°C during the day (RMSE) and 0.4°C at night in the wet season. In the dry season, these values are 1.4°C during the day and 1°C at night. The standard deviation of the modeled lapse rate during the wet season is 0.01°C, for the measured lapse rate it is 0.2°C. During the dry season the standard deviation of the modeled lapse rate is 0.1°C, for the measured lapse rate it is 0.4°C. Based on these findings, the altitude and time-of-day-dependent temperature gradients *microclima* model outputs are expected to be less pronounced and will be subject to substantially lower fluctuations.

### Downscaling results

**Fig 8:**
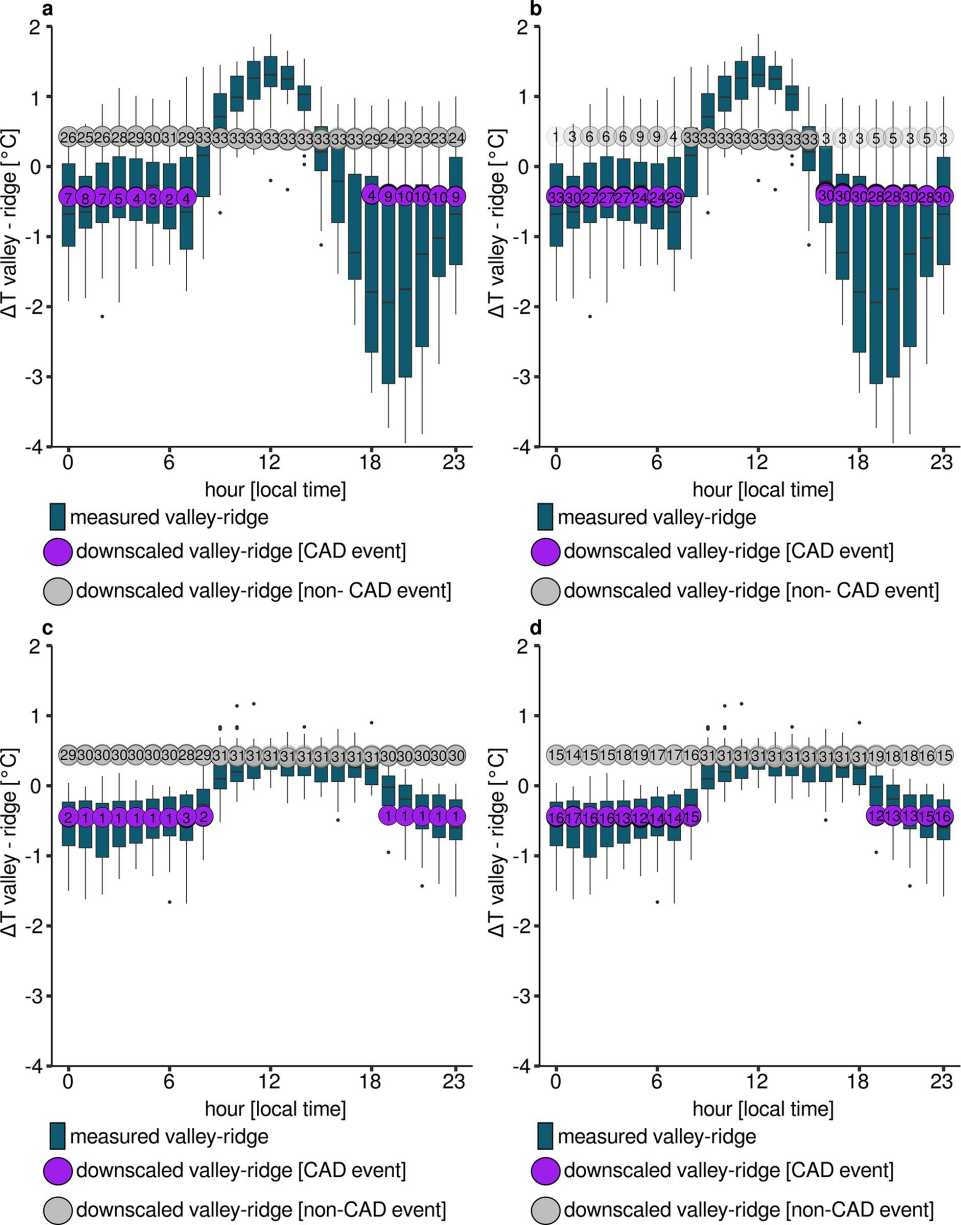
Modeled temperature differences obtained from downscaling results in both seasons with default and modified settings compared to measurements. **a** Dry season with default settings. **b** Dry season with modified settings. **c** Wet season with default settings. **d** Wet season with modified settings. Modified settings incorporate an emissivity threshold of 0.9, enlarged CAD detection times during the dry season and a wind threshold of 0.9 m/s.

The model outputs for the model run extent with maximum radius (28.800 meters) and a maximum threshold of merging adjacent basins of 100 meters are presented below (**Table 3**, **Fig 8**). Based on the applications of the *microclima* functions with default settings, a large number of CAD events are not detected in the dry season. The daytime calculations are correct in their dimension, as the valley station is modeled as warmer than the ridge station. However, the diurnal course of temperatures is not modeled correctly. First, the amplitude of the temperature differences between valley and ridge station is not represented strongly enough. Second, the transitions between CAD events and non-CAD events are very sharp. A cooling in which the temperature inversion develops over several hours (between 3 and 5 p.m.) is not recognizable in the values modeled with *microclima*. In particular, the strong temperature gradients at midday and at the beginning of the cold air accumulation in the valley between 5 and 8 p.m. are not correctly reproduced. In the early morning hours, the temperature gradient between the measuring points is constant and at a similar level, just like in the microclimate output (**Fig 8A and 8B**).

**Table 3:** RMSE between measured and modeled temperature gradients between valley and ridge position dependending on season, time of day and subset of data. Please note that measured values change during the modified dry season since the hours of 4 and 5 p.m. are added to the nighttime class.

season	subset	model settings	ΔT_valley-ridge_^measured^ [°C]	ΔT_valley-ridge_^modeled^ [°C]	RMSE [°C]
dry	full	default	-0.3	0.3	1.3
dry	day	default	0.6	0.4	0.9
dry	night	default	-0.8	0.3	1.5
dry	full	modified	-0.3	-0.1	1.1
dry	day	modified	1.0	0.4	0.8
dry	night	modified	-0.7	-0.2	1.2
wet	full	default	-0.2	0.4	0.8
wet	day	default	0.3	0.4	0.3
wet	night	default	-0.4	0.4	0.9
wet	full	modified	-0.2	0.2	0.6
wet	day	modified	0.3	0.4	0.3
wet	night	modified	-0.4	0.1	0.7

During the wet season the majority of inversions are not correctly classified as CAD events with both default and modified settings. In contrast to the dry season, however, the amplitude of the temperature difference is much better represented by the *microclima* functions (**Fig 8C and 8D**).

### The effect of basin merges

**Fig 9:**
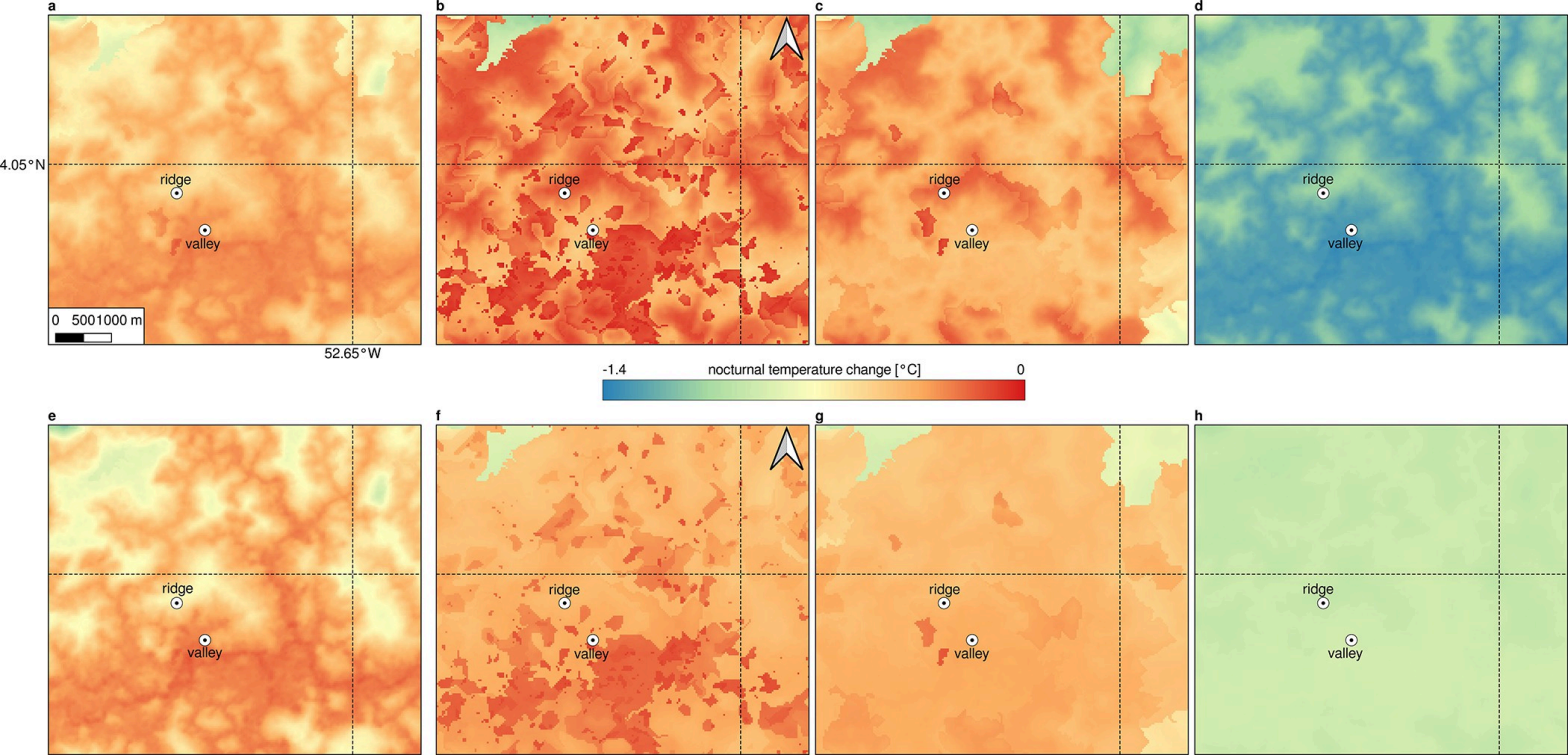
Different thresholds of basin merges and their effect on spatial patterns in long-term mean outputs from the microclima functions. **a** Dry season with default basins and default settings (basins not visible due to only few CAD event detections). **b** Dry season with default basins and modified model settings. **c** Dry season with a basin merge threshold of 10 meters. **d** Dry season with a basin merge threshold of 100 meters. **e** Wet season with default basins and default settings. **f** Wet season with default basins and modified model settings. **g** Wet season with a basin merge threshold of 10 meters and modified model settings. **g** Wet season with a basin merge threshold of 100 meters and modified model settings. Modified model settings include an emissivity threshold of .90, a wind threshold of 0.9 m/s and an extended time for possible CAD event detection to 4 and 5 pm during the dry season.

In addition to the pre-defining values of the moist adiabatic lapse rate, the model run extent and the CAD condition detection, the settings regarding CAD basins are crucial for the results obtained with the *microclima* functions. If no CAD events are detected, the basin layer used is not visible in the results (**Fig 9A**). If basins are not merged, extensive artifacts in the spatial representation of the expected temperature differences due to CAD flows are observed (**Fig 9B**). Merging adjacent basins with a threshold value of 10 meters allows the coarse terrain structure to be punctured more clearly, whereby high elevational gradients in particular become visible (**Fig 9B and 6C**), for example in the area surrounding the ridge station. With a merging by means of a threshold value of 100m, all grid cells are combined into one basin. For this reason, the fine-scale terrain structure clearly appears in the result of the predicted CAD-related cooling without artifacts (**Fig 9D**). This means that all basins are interdependent and cold air can arrive anywhere in the model area. There are therefore no barriers between individual basins and all basins exchange cold air with each other. The cooling at each point in the study area then depends on the highest elevation information, which is included in the calculation. During the wet season (**Fig 9E**-**9H**) the effects of basin merges is not as evident as during the dry season since far less CAD events are detected affecting the long-term spatial patterns in temperature.

## Discussion

In this study, the algorithms presented in the *microclima* R package were analyzed regarding their usability in the South American tropical lowland forests. Our findings reveal that the *microclima* model is not fully capable of correctly modeling the temperatures necessary for niche formation for canopy epiphytes in tropical lowland forests. In particular, the model shows shortcomings in the application in the study area with regard to the detection of inversions and their classification as CAD events and in the modeling of temperature gradients, whether during the day due to solar radiation or at night due to CAD flows. In some cases, as in the dry season, these shortcomings can be reduced by simple adjustments such as modifying threshold values for emissivity, wind speed and the times at which CAD event detection is possible. For the wet season, however, it is not possible to improve the model performance satisfactorily with these simple adjustments. In particular, CAD event detection cannot be based on emissivity due to the constant dense cloud cover in the study area. For the correctly classified CAD events, it is necessary to make adjustments to *microclima* with regard to the temperature amplitude. The modeled temperature gradients were increased in the present study by greatly enlarging the study area and unlimited merging of the cold air basins. This is not recommended for the general application of the model. In *microclima*, the lapse rate for the calculated temperatures and therefore temperature amplitudes is the key driver. When using the lapse rate, adjustments must be made in order to correctly model the warming effects during day [42] and, in particular, the cooling effects caused by CAD flows. If the intensity of the CAD-related effects is not modeled correctly, the cooling may be too weak. As a result, the temperature may not drop below the dew point temperature required for FLS formation within the valleys [9, 41].

Contrary to the results presented here, the *microclima* model has proven to be able to provide solid performance when modeling high-resolution temperature data. However, the model was used in both studies in temperate zones [25, 30]. The effects of diurnal warming and nocturnal CAD flows are considerably lower in the areas investigated so far, so that these can be correctly mapped by the functions implemented in *microclima*. Recalibration of the *microclima* model parameters is required for use in the tropics or outside the area for which *microclima* was developed and calibrated. Existing downscaling algorithms should be intensively tested for their performance for each study area and targeted spatio-temporal resolution and adapted where necessary. Depending on the location, different local factors such as topography and land cover type can influence the downscaling procedures. Therefore, the most sensitive local factors need to be identified for each study area. We recommend validation with more observations, especially in remote and normally data-poor regions, which could also be used to extend the present study in the future. Long-term measurements for calibration and validation of downscaling approaches can be particularly helpful, as they provide good coverage of both many data points and seasonal phenomena such as weather extremes, which are expected to increase in the future. For the continuous temporal modeling of CAD flows, we also recommend implementing temporal accumulations of cold air masses as in KLAM21.
